# Supplementary material for: Functional Frailty, Dietary Intake, and Risk of Malnutrition. Are Nutrients Involved in Muscle Synthesis the Key for Frailty Prevention?
Source: Nutrients. 2021 Apr 8;13(4):1231. doi: 10.3390/nu13041231 (PMC8068284; doi:10.3390/nu13041231)
Supplement: Supplementary file 1 [file nutrients-13-01231-s001.pdf]

Supplementary material, Table S1. Data about graphs; ANCOVA considering robust as reference group

**Nutrient intake**	**Robust**	**Pre-Frail**	**Frail**
Carbohydrates (g/day)	Ref	26.9±14.0	**44.9±16.4**
Protein (g/day)	Ref	**-11.0±5.1**	**-13.4±6.0**
Total fat (g/day)	Ref	-8.6±5.4	**-13.0±6.4**
Alcohol (g/day)	Ref	2.0±2.7	-1.3±3.1
w-3 (g/day)	Ref	-0.4±0.4	**-1.1±0.4**
w-6 (g/day)	Ref	1.3±1.8	-1.0±2.1
MUFA (g/day)	Ref	-2.6±3.8	-4.6±4.4
PUFA (g/day)	Ref	1.0±1.0	-1.9±2.4
SFA (g/day)	Ref	**-6.2±2.2**	**-6.6±2.6**
Retinol equivalents (µg/day)	Ref	-125.8±206.7	-267.9±243.1
Vitamin D (µg/day)	Ref	-1.8±1.1	**-3.2±1.3**
Vitamin E (mg/day)	Ref	0.6±0.5	0.7±1.0
Ascorbic acid (C) (mg/day)	Ref	-33.3±35.7	-69.3±42.0
Tiamin (B1) (mg/day)	Ref	-0.2±0.5	-0.2±0.3
Riboflavin (B2) (mg/day)	Ref	-0.2±0.3	-0.3±0.2
Niacin equivalents (B3) (mg/day)	Ref	**-5.5±2.7**	**-7.4±3.2**
Pyridoxine (B6) (mg/day)	Ref	-0.1±0.2	**-0.4±0.2**
Folic acid (B9) (µg/day)	Ref	-10.7±36.9	-55.9±43.4
Cobalamin (B12) (µg/day)	Ref	-1.4±1.4	-2.4±1.6
Calcium (mg/day)	Ref	-172.1±107.1	-154.3±126.1
Iron (mg/day)	Ref	-0.3±1.0	-0.3±1.3
Sodium (mg/day)	Ref	-258.7±172.1	-78.0±202.4
Magnesium (mg/day)	Ref	-17.5±25.4	-18.4±29.9
Potassium (mg/day)	Ref	-114.8±315.3	-536.3±370.9
Iodine (µg/day)	Ref	32.0±0.5	68.5±56.8
Selenium (µg/day)	Ref	-9.5±7.6	-10.2±8.9
Zinc (mg/day)	Ref	-1.0±0.7	-0.7±0.8
Phosphorus (mg/day)	Ref	**-265.5±118.7**	-237.7±139.7

* Bold letter represents statistically significant difference comparing with the robust.
